# Author Correction: Low dose rate γ-irradiation protects fruit fly chromosomes from double strand breaks and telomere fusions by reducing the esi-RNA biogenesis factor *Loquacious*

**DOI:** 10.1038/s42003-022-03984-8

**Published:** 2022-09-29

**Authors:** A. Porrazzo, F. Cipressa, A. De Gregorio, C. De Pittà, G. Sales, L. Ciapponi, P. Morciano, G. Esposito, M. A. Tabocchini, G. Cenci

**Affiliations:** 1 Dipartimento di Biologia e Biotecnologie “C. Darwin”, Sapienza Università di Roma, Rome, Italy; 2 Fondazione Cenci Bolognetti/ Istituto Pasteur Italia, Rome, Italy; 3 Centro Studi e Ricerche “Enrico Fermi”, Rome, Italy; 4 Department of Ecological and Biological Sciences, Università degli Studi della Tuscia, Viterbo, Italy; 5 Dipartimento di Biologia, Università di Padova, Padua, Italy; 6 INFN-Laboratori Nazionali del Gran Sasso, 67100 Assergi, Italy; 7 Istituto Superiore di Sanita‘ ISS, Rome, Italy; 8 INFN-Roma 1, Rome, Italy

**Keywords:** Genomic instability, Developmental biology

Correction to: *Communications Biology* 10.1038/s42003-022-03885-w, published online 03 September 2022.

In the original version of the Article, the affiliation ‘Department of Ecological and Biological Sciences, Università degli Studi della Tuscia, Viterbo, Italy’ for F. Cipressa was missing. The original article has been corrected.

