# Supplementary material for: Low dose rate γ-irradiation protects fruit fly chromosomes from double strand breaks and telomere fusions by reducing the esi-RNA biogenesis factor Loquacious
Source: Commun Biol. 2022 Sep 3;5:905. doi: 10.1038/s42003-022-03885-w (PMC9440893; doi:10.1038/s42003-022-03885-w)
Supplement: Supplementary file 3 — Description of Additional Supplementary Files [file 42003_2022_3885_MOESM3_ESM.pdf]

Description of Additional Supplementary Files

File name: Supplementary Data 1-6
Description:

Supplementary Data 1: Differentially expressed genes between 0.4Gy LDR + 10Gy irradiated vs. unirradiated larval male brains.

Supplementary Data 2: Differentially expressed genes between 0.4Gy LDR + 10Gy vs. 10Gy irradiated larval male brains.

Supplementary Data 3: Differentially expressed genes between 0.4Gy LDR + 10Gy vs. 0.4Gy LDR irradiated larval male brains.

Supplementary Data 4: Differentially expressed genes between 10Gy irradiated vs. unirradiated larval male brains.

Supplementary Data 5: Differentially expressed genes between 10Gy vs. 0.4Gy LDR irradiated larval male brains.

Supplementary Data 6: RNA processing biological process.
