# Supplementary material for: Low dose rate γ-irradiation protects fruit fly chromosomes from double strand breaks and telomere fusions by reducing the esi-RNA biogenesis factor Loquacious
Source: Commun Biol. 2022 Sep 3;5:905. doi: 10.1038/s42003-022-03885-w (PMC9440893; doi:10.1038/s42003-022-03885-w)
Supplement: Supplementary file 4 — Supplementary Data 1-6 [file 42003_2022_3885_MOESM4_ESM.pdf]

Low dose rate γ-irradiation protects fruit fly chromosomes from double strand breaks and telomere fusions by reducing the esi-RNA biogenesis factor *Loquacious*

Porrazzo A., Cipressa F., De Gregorio A. , De Pittà C., Sales G., Morciano P., Esposito G. Ciapponi L., Tabocchini M.A., Cenci G.

Supplementary Data

Supplementary Data 1. Differentially expressed genes between 0.4Gy LDR + 10Gy irradiated *vs*. unirradiated larval male brains.

A list of 106 differentially expressed genes (94 up-regulated and 12 down-regulated) from 0.4Gy LDR + 10Gy (D) *vs*. unirradiated (A) comparison considering a 5% false discovery rate. In the last column are represented the expression levels of each transcript calculated as Log2(D/A).

FlyBase ID	Enrez ID	Description	log2(D/A)
agt	40816	O-6-alkylguanine-DNA alkyltransferase(agt)	0.62
Amnionless	33199	nogaster Amnionless ortholog(Amnionless)	0.64
AOX1	41894	Aldehyde oxidase 1(AOX1)	1.33
CG10005	41451	CG10005 gene product from transcript CG10005-RA(CG10005)	0.76
CG10638	39424	CG10638 gene product from transcript CG10638-RD(CG10638)	0.66
CG11131	40511	CG11131 gene product from transcript CG11131-RB(CG11131)	0.37
CG11897	43450	CG11897 gene product from transcript CG11897-RB(CG11897)	1.01
CG12171	40690	CG12171 gene product from transcript CG12171-RA(CG12171)	0.53
CG12224	41453	CG12224 gene product from transcript CG12224-RC(CG12224)	0.63
CG12264	34613	CG12264 gene product from transcript CG12264-RA(CG12264)	0.72
CG1299	38496	CG1299 gene product from transcript CG1299-RA(CG1299)	0.65
CG13067	39785	CG13067 gene product from transcript CG13067-RA(CG13067)	0.55
CG13705	38587	CG13705 gene product from transcript CG13705-RA(CG13705)	0.40
CG14419	31276	CG14419 gene product from transcript CG14419-RA(CG14419)	0.38
CG15784	31461	CG15784 gene product from transcript CG15784-RB(CG15784)	1.12
CG1582	32021	CG1582 gene product from transcript CG1582-RB(CG1582)	0.51
CG17104	34465	CG17104 gene product from transcript CG17104-RB(CG17104)	0.67
CG1809	35981	CG1809 gene product from transcript CG1809-RA(CG1809)	0.00
CG18547	41452	CG18547 gene product from transcript CG18547-RA(CG18547)	1.48
CG3008	33693	CG3008 gene product from transcript CG3008-RA(CG3008)	0.85
CG31869	34490	CG31869 gene product from transcript CG31869-RC(CG31869)	0.59
CG32071	39246	CG32071 gene product from transcript CG32071-RA(CG32071)	0.24
CG32603	318109	CG32603 gene product from transcript CG32603-RA(CG32603)	0.18
CG33158	39834	CG33158 gene product from transcript CG33158-RB(CG33158)	0.51
CG34417	31591	CG34417 gene product from transcript CG34417-RI(CG34417)	0.39
CG3448	39094	CG3448 gene product from transcript CG3448-RB(CG3448)	0.78
CG4115	41499	CG4115 gene product from transcript CG4115-RA(CG4115)	0.80
CG42326	35838	CG42326 gene product from transcript CG42326-RE(CG42326)	0.63
CG43427	40583	CG43427 gene product from transcript CG43427-RP(CG43427)	0.33
CG43693	39231	CG43693 gene product from transcript CG43693-RD(CG43693)	-0.41
CG5059	40278	CG5059 gene product from transcript CG5059-RD(CG5059)	-0.32
CG5205	41891	CG5205 gene product from transcript CG5205-RA(CG5205)	0.55
CG5955	40268	CG5955 gene product from transcript CG5955-RA(CG5955)	0.74
CG6512	39922	CG6512 gene product from transcript CG6512-RB(CG6512)	0.45
CG6901	41977	CG6901 gene product from transcript CG6901-RA(CG6901)	0.61
CG7627	34148	CG7627 gene product from transcript CG7627-RB(CG7627)	0.53
CG8064	42196	CG8064 gene product from transcript CG8064-RA(CG8064)	0.30
CG9090	37297	CG9090 gene product from transcript CG9090-RA(CG9090)	0.58
CG9297	41688	CG9297 gene product from transcript CG9297-RC(CG9297)	0.62
CG9411	32392	CG9411 gene product from transcript CG9411-RA(CG9411)	0.61
CG9759	41661	CG9759 gene product from transcript CG9759-RB(CG9759)	0.40
CHKov2	43068	CG10675 gene product from transcript CG10675-RA(CHKov2)	0.68
Cht6	31935	CG43374 gene product from transcript CG43374-RK(Cht6)	0.55
Corp	31764	Companion of reaper(Corp)	0.66
Cpr100A	43657	Cuticular protein 100A(Cpr100A)	0.43
CR43144	19834780	ncRNA(CR43144)	0.74
CR44272	19835298	ncRNA(CR44272)	-0.61
CR44430	19835618	ncRNA(CR44430)	0.70
CR44751	19834965	ncRNA(CR44751)	0.46

CR46003	26067324	ncRNA(CR46003)	-0.32
cv-2	45280	crossveinless 2(cv-2)	0.80
dally	39013	division abnormally delayed(dally)	0.32
Dif	35045	Dorsal-related immunity factor(Dif)	0.46
dp	36461	DP transcription factor(Dp)	0.41
dyl	38531	dusky-like(dyl)	0.92
E(spl)m5-HLH	43158	Enhancer of split m5. helix-loop-helix(E(spl)m5-HLH)	-0.63
E(spl)m6-BFM	43159	Enhancer of split m6. Bearded family member(E(spl)m6-BFM)	-0.59
E(spl)mgamma-HLH	43151	Enhancer of split mgamma. helix-loop-helix(E(spl)mgamma-HLH)	-0.60
Ect4	38895	Ectoderm-expressed 4(Ect4)	0.36
egr	36054	eiger(egr)	0.35
form3	3346238	formin 3(form3)	0.35
Gadd45	35646	CG11086 gene product from transcript CG11086-RA(Gadd45)	0.84
Gclc	53581	Glutamate-cysteine ligase catalytic subunit(Gclc)	0.50
Gcn5	39431	Gcn5 ortholog(Gcn5)	0.32
GstD3	48336	Glutathione S transferase D3(GstD3)	0.56
GstE5	37110	Glutathione S transferase E5(GstE5)	0.60
GstE6	37111	Glutathione S transferase E6(GstE6)	0.95
hid	40009	head involution defective(hid)	0.89
Hmgcr	42803	HMG Coenzyme A reductase(Hmgcr)	0.47
HmgZ	37480	HMG protein Z(HmgZ)	-0.44
Hsc70-5	36583	Heat shock protein cognate 5(Hsc70-5)	0.29
Hsp60	32045	Heat shock protein 60(Hsp60)	0.36
Ilp8	39909	Insulin-like peptide 8(Ilp8)	1.28
Irbp	117419	Inverted repeat-binding protein(Irbp)	0.72
JhI-26	36819	Juvenile hormone-inducible protein 26(JhI-26)	0.70
Ku80	34930	CG18801 gene product from transcript CG18801-RA(Ku80)	0.67
l(3)72Ab	39737	lethal (3) 72Ab(l(3)72Ab)	0.31
magu	36048	CG2264 gene product from transcript CG2264-RA(magu)	-0.47
Mdr49	36428	Multi drug resistance 49(Mdr49)	0.70
mfas	41455	midline fasciclin(mfas)	0.27
Mocs1	39238	Molybdenum cofactor synthesis 1 ortholog(Mocs1)	0.59
mre11	34565	meiotic recombination 11(mre11)	0.55
MRP	34686	Multidrug-Resistance like Protein 1(MRP)	0.75
mus205	47186	mutagen-sensitive 205(mus205)	0.54
nerfin-1	44786	nervous fingers 1(nerfin-1)	-0.21
Nplp4	50190	Neuropeptide-like precursor 4(Nplp4)	1.09
PEK	40653	pancreatic eIF-2alpha kinase(PEK)	0.35
pic	41611	piccolo(pic)	0.27
POSH	36990	Plenty of SH3s(POSH)	0.30
Pvf2	33994	PDGF- and VEGF-related factor 2(Pvf2)	0.49
RnrL	34392	Ribonucleoside diphosphate reductase large subunit(RnrL)	0.34
roX1	3772376	RNA on the X 1(roX1)	-0.31
rpr	40015	reaper(rpr)	0.73
scaf	35505	scarface(scaf)	0.61
scb	36692	scab(scb)	0.65
sda	44359	slamdance(sda)	0.36
snoRNA:Psi18S-525c	5740385	ncRNA(snoRNA:Psi18S-525c)	-0.58
spn-E	41919	spindle E(spn-E)	0.85
Sulf1	53437	Sulfated(Sulf1)	0.56
Traf4	33638	TNF-receptor-associated factor 4(Traf4)	0.43
Ugt86Da	53510	CG18578 gene product from transcript CG18578-RA(Ugt86Da)	0.79
Ugt86Di	53502	CG6658 gene product from transcript CG6658-RB(Ugt86Di)	0.94
Vinc	31201	Vinculin(Vinc)	0.38
wnd	40143	wallenda(wnd)	0.27
Xrp1	42267	CG17836 gene product from transcript CG17836-RB(Xrp1)	0.53
zip	38001	zipper(zip)	0.31

Supplementary Data 2. Differentially expressed genes between 0.4Gy LDR + 10Gy *vs*. 10Gy irradiated larval male brains.

A list of 107 differentially expressed genes (42 up-regulated and 65 down-regulated) from 0.4Gy LDR + 10Gy (D) *vs*. 10Gy comparison considering a 5% false discovery rate. In the last column are represented the expression levels of each transcript calculated as Log_2 (D/B).

FlyBase ID	Entrez ID	Description	Log2(D/B)
Aats-Ile	45785	Isoleucyl-tRNA synthetase [Source:FlyBase	0.32
Aldh	34256	Aldehyde dehydrogenase [Source:FlyBase	-0.43
Amnionless	33199	Amnionless ortholog [Source:FlyBase	0.51
Aox1	41894	Aldehyde oxidase 1 [Source:FlyBase	0.48
Aurb	34504	aurora B [Source:FlyBase	-0.51
Beat-Iiic	35037	beat-IIIc [Source:FlyBase	-0.34
Bwa	250736	brain washing [Source:FlyBase	-0.39
Cand1	34403	Cullin-associated and neddylation-dissociated 1 [Source:FlyBase	0.28
CG10005	41451	RE59626p [Source:UniProtKB/TrEMBL	0.54
CG11151	32335	GEO07753p1 [Source:UniProtKB/TrEMBL	-0.49
CG12194	33685	Uncharacterized protein. isoform B [Source:UniProtKB/TrEMBL	-0.28
CG12325	36144	LD10780p [Source:UniProtKB/TrEMBL	0.40
CG12499	42637	NA	0.37
CG13185	36268	NA	0.39
CG13784	34003	Uncharacterized protein. isoform E [Source:UniProtKB/TrEMBL	-0.29
CG17680	37071	Essential MCU regulator. mitochondrial [Source:UniProtKB/Swiss-Prot	-0.39
CG1896	43738	CG1896 [Source:UniProtKB/TrEMBL	-0.44
CG30349	35885	FI03455p [Source:UniProtKB/TrEMBL	0.32
CG31683	261623	BcDNA.GH02384 [Source:UniProtKB/TrEMBL	-0.55
CG31869	34490	Uncharacterized protein. isoform A [Source:UniProtKB/TrEMBL	0.40
CG32026	317829	GH12815p [Source:UniProtKB/TrEMBL	0.43
CG32318	2768976	RT07324p [Source:UniProtKB/TrEMBL	-0.52
CG32344	326208	LD28101p [Source:UniProtKB/TrEMBL	0.28
CG33158	39834	NA	0.42
CG34159	34353	GEO11246p1 [Source:UniProtKB/TrEMBL	-0.43
CG34232	5740195	LP22624p [Source:UniProtKB/TrEMBL	-0.62
CG3835	31184	CG3835. isoform A [Source:UniProtKB/TrEMBL	-0.49
CG40228	5740664	Transcription elongation factor 1 homolog [Source:UniProtKB/Swiss-Prot	-0.37
CG41128	3355108	MICOS complex subunit MIC10 [Source:UniProtKB/TrEMBL	-0.55
CG42231	7354432	NA	-0.58
CG42362	7354407	IP15503p1 [Source:UniProtKB/TrEMBL	0.54
CG43349	12797873	Uncharacterized protein. isoform A [Source:UniProtKB/TrEMBL	-0.16
CG4554	37570	CG4554 [Source:UniProtKB/TrEMBL	0.40
CG5205	41891	CG5205 [Source:UniProtKB/TrEMBL	0.39
CG6388	34656	Probable tRNA (guanine(26)-N(2))-dimethyltransferase [Source:UniProtKB/Swiss-Prot	0.38
CG7607	39263	CG7607 [Source:UniProtKB/TrEMBL	-0.48
CG7656	39691	CG7656. isoform D [Source:UniProtKB/TrEMBL	-0.35
CG8064	42196	CG8064 [Source:UniProtKB/TrEMBL	0.36
CG8939	32568	Putative rRNA methyltransferase [Source:UniProtKB/TrEMBL	0.47
Clbn	43018	Caliban [Source:FlyBase	0.47
Cr33987	3885608	ncRNA(CR33987)	-0.58
Cr43900	14462580	ncRNA(CR43900)	-0.65
Cr44272	19835298	ncRNA(CR44272)	-0.56
Cr45168	19835216	ncRNA(CR45168)	-0.75
Cr45820	26067148	ncRNA(CR45820)	0.58
Cr45908	26067236	ncRNA(CR45908)	-0.62
Ctpsyn	39645	CTP synthase [Source:FlyBase	0.32
Cv-2	45280	crossveinless 2 [Source:FlyBase	0.40
Den1	36339	Deneddylase 1 [Source:FlyBase	-0.29
Dnaj-60	37869	DnaJ-like-60 [Source:FlyBase	-0.57
Drat	35687	Death resistor Adh domain containing target [Source:FlyBase	-0.37
Eif4g	43839	eukaryotic translation initiation factor 4G [Source:FlyBase	0.29
Gs1	33172	Glutamine synthetase 1 [Source:FlyBase	-0.35
Hey	35764	Hairy/E(spl)-related with YRPW motif [Source:FlyBase	-0.40
His2b:CG33868	3772265	His2B:CG33868 [Source:FlyBase	0.23
Ifc	33836	infertile crescent [Source:FlyBase	-0.27
Kra	40680	krasavietz [Source:FlyBase	0.43
Kug	40191	kugelei [Source:FlyBase	0.34
L(2)35df	48782	lethal (2) 35Df [Source:FlyBase	0.30
L(2)K09022	33960	lethal (2) k09022 [Source:FlyBase	0.40
L(3)72ab	39737	lethal (3) 72Ab [Source:FlyBase	0.28
Lk6	44672	Lk6 [Source:FlyBase	-0.25
Loqs	34751	loquacious [Source:FlyBase	-0.48
Med7	41288	Mediator complex subunit 7 [Source:FlyBase	-0.50
Mpc1	42268	Mitochondrial pyruvate carrier [Source:FlyBase	-0.46
Mrp	34686	Multidrug-Resistance like Protein 1 [Source:FlyBase	0.42
Mrpl33	50381	mitochondrial ribosomal protein L33 [Source:FlyBase	-0.50
Mrps21	318249	mitochondrial ribosomal protein S21 [Source:FlyBase	-0.61

Mrps28	37136	mitochondrial ribosomal protein S28 [Source:FlyBase	**-0.49**
Mys45a	35925	Mystery 45A [Source:FlyBase	**0.39**
Nplp4	50190	Neuropeptide-like precursor 4 [Source:FlyBase	**0.69**
Pic	41611	piccolo [Source:FlyBase	**0.25**
Pink1	31607	PTEN-induced putative kinase 1 [Source:FlyBase	**-0.38**
Pis	32506	Phosphatidylinositol synthase [Source:FlyBase	**-0.40**
Posh	36990	Plenty of SH3s [Source:FlyBase	**0.34**
Ptr	35546	Patched-related [Source:FlyBase	**-0.47**
Rca1	33959	Regulator of cyclin A1 [Source:FlyBase	**-0.42**
Rheb	117332	Ras homolog enriched in brain ortholog (H. sapiens) [Source:FlyBase	**-0.38**
Rme-8	35939	Receptor mediated endocytosis 8 [Source:FlyBase	**0.26**
Rnasep:Rna	3772418	Ribonuclease P RNA [Source:FlyBase	**-0.56**
Roc2	36246	Regulator of cullins 2 [Source:FlyBase	**-0.45**
Rpi1	36617	RNA polymerase I subunit [Source:FlyBase	**0.34**
Rpl35	31483	Ribosomal protein L35 [Source:FlyBase	**-0.46**
Rpl36	31009	Ribosomal protein L36 [Source:FlyBase	**-0.50**
Rpl36a	34098	Ribosomal protein L36A [Source:FlyBase	**-0.56**
Rpl38	3355144	Ribosomal protein L38 [Source:FlyBase	**-0.51**
Rpl39	37849	Ribosomal protein L39 [Source:FlyBase	**-0.57**
Rps29	41200	Ribosomal protein S29 [Source:FlyBase	**-0.46**
Scarna:Meu5-C46	3772530	small Cajal body-specific RNA : MeU5-C46 [Source:FlyBase	**-0.55**
Sirup	34089	Starvation-upregulated protein [Source:FlyBase	**-0.62**
Skl	40016	sickle [Source:FlyBase	**-0.52**
Snorna:Psi18s-1389a	5740842	snoRNA:Psi18S-1389a [Source:FlyBase	**-0.63**
Snorna:Psi28s-1135f	5740107	snoRNA:Psi28S-1135f [Source:FlyBase	**-0.58**
Snorna:Psi28s-1175a	5740342	snoRNA:Psi28S-1175a [Source:FlyBase	**-0.62**
Snorna:Psi28s-1180	3771901	snoRNA:Psi28S-1180 [Source:FlyBase	**-0.58**
Snorna:Psi28s-2626	5740708	snoRNA:Psi28S-2626 [Source:FlyBase	**-0.62**
Snorna:Psi28s-3342	3771886	snoRNA:Psi28S-3342 [Source:FlyBase	**-0.69**
Snrna:U2:34abb	3771927	small nuclear RNA U2 at 34ABb [Source:FlyBase	**-0.70**
Snrna:U4atac:82e	12798231	small nuclear RNA U4atac at 82E [Source:FlyBase	**-0.62**
Snrna:U6:96aa	3772327	small nuclear RNA U6 at 96Aa [Source:FlyBase	**-0.56**
Sra	47384	sarah [Source:FlyBase	**-0.31**
Su(Ste):Cr42426	7354460	Su(Ste):CR42426 [Source:FlyBase	**0.68**
Su(Ste):Cr42430	7354464	Su(Ste):CR42430 [Source:FlyBase	**0.63**
Sulf1	53437	Sulfated [Source:FlyBase	**0.40**
Tbp	37476	TATA binding protein [Source:FlyBase	**-0.45**
Ugt86di	53502	Ugt86Di [Source:FlyBase	**0.61**
Zip	38001	zipper [Source:FlyBase	**0.25**

Supplementary Data 3. Differentially expressed genes between 0.4Gy LDR + 10Gy *vs*. 0.4Gy LDR irradiated larval male brains.

A list of 83 differentially expressed genes (72 up-regulated and 11 down-regulated) from 0.4Gy LDR + 10Gy (D) *vs*. 0.4Gy LDR (C) comparison considering a 5% false discovery rate. In the last column are represented the expression levels of each transcript calculated as Log2(D/C).

FlyBase ID	Entrez ID	Description	Log2(D/C)
agt	40816	O-6-alkylguanine-DNA alkyltransferase(agt)	0.60
AOX1	41894	Aldehyde oxidase 1(AOX1)	1.22
Bace	34182	beta-site APP-cleaving enzyme(Bace)	-0.35
baz	32703	bazooka(baz)	0.43
CG10005	41451	CG10005 gene product from transcript CG10005-RA(CG10005)	0.75
CG10445	40938	CG10445 gene product from transcript CG10445-RD(CG10445)	0.62
CG10570	50466	CG10570 gene product from transcript CG10570-RC(CG10570)	-0.64
CG10638	39424	CG10638 gene product from transcript CG10638-RD(CG10638)	0.74
CG11897	43450	CG11897 gene product from transcript CG11897-RB(CG11897)	1.04
CG12264	34613	CG12264 gene product from transcript CG12264-RA(CG12264)	0.75
CG15784	31461	CG15784 gene product from transcript CG15784-RB(CG15784)	1.04
CG1582	32021	CG1582 gene product from transcript CG1582-RB(CG1582)	0.51
CG17104	34465	CG17104 gene product from transcript CG17104-RB(CG17104)	0.71
CG1809	35981	CG1809 gene product from transcript CG1809-RA(CG1809)	-0.01
CG18213	42055	CG18213 gene product from transcript CG18213-RD(CG18213)	0.65
CG18547	41452	CG18547 gene product from transcript CG18547-RA(CG18547)	1.36
CG2064	35708	CG2064 gene product from transcript CG2064-RA(CG2064)	0.58
CG3008	33693	CG3008 gene product from transcript CG3008-RA(CG3008)	0.88
CG30269	37600	CG30269 gene product from transcript CG30269-RB(CG30269)	-0.02
CG31869	34490	CG31869 gene product from transcript CG31869-RC(CG31869)	0.67
CG33158	39834	CG33158 gene product from transcript CG33158-RB(CG33158)	0.50
CG33502	2768875	CG33502 gene product from transcript CG33502-RA(CG33502)	0.25
CG3448	39094	CG3448 gene product from transcript CG3448-RB(CG3448)	0.78
CG42304	38332	CG42304 gene product from transcript CG42304-RA(CG42304)	0.10
CG43427	40583	CG43427 gene product from transcript CG43427-RP(CG43427)	0.31
CG43693	39231	CG43693 gene product from transcript CG43693-RD(CG43693)	-0.40
CG5205	41891	CG5205 gene product from transcript CG5205-RA(CG5205)	0.57
CG5955	40268	CG5955 gene product from transcript CG5955-RA(CG5955)	0.76
CG7627	34148	CG7627 gene product from transcript CG7627-RB(CG7627)	0.64
CG8064	42196	CG8064 gene product from transcript CG8064-RA(CG8064)	0.29
CHKov2	43068	CG10675 gene product from transcript CG10675-RA(CHKov2)	0.62
Corp	31764	Companion of reaper(Corp)	0.65
Cpr78E	40408	Cuticular protein 78E(Cpr78E)	-0.76
CR43144	19834780	ncRNA(CR43144)	1.05
CR44751	19834965	ncRNA(CR44751)	0.46
Cul2	35420	Cullin 2(Cul2)	0.54
cv-2	45280	crossveinless 2(cv-2)	0.59
Cyp6a20	36664	CG10245 gene product from transcript CG10245-RB(Cyp6a20)	0.61
dally	39013	division abnormally delayed(dally)	0.35
Dif	35045	Dorsal-related immunity factor(Dif)	0.52
dyl	38531	dusky-like(dyl)	0.75
E(spl)m5-HLH	43158	Enhancer of split m5. helix-loop-helix(E(spl)m5-HLH)	-0.57
Ect4	38895	Ectoderm-expressed 4(Ect4)	0.32
egr	36054	eiger(egr)	0.43
Gadd45	35646	CG11086 gene product from transcript CG11086-RA(Gadd45)	0.77
Gcn5	39431	Gcn5 ortholog(Gcn5)	0.41
GstD3	48336	Glutathione S transferase D3(GstD3)	0.47
GstE6	37111	Glutathione S transferase E6(GstE6)	0.92
hid	40009	head involution defective(hid)	0.88
Hmgcr	42803	HMG Coenzyme A reductase(Hmgcr)	0.47
Ilp8	39909	Insulin-like peptide 8(Ilp8)	1.23
Invadolysin	49580	CG3953 gene product from transcript CG3953-RA(Invadolysin)	0.28
Irbp	117419	Inverted repeat-binding protein(Irbp)	0.78
Ku80	34930	CG18801 gene product from transcript CG18801-RA(Ku80)	0.78
l(3)72Ab	39737	lethal (3) 72Ab(l(3)72Ab)	0.35
lig3	41518	DNA ligase III(lig3)	0.46
mahj	37462	mahjong(mahj)	0.29
Mocs1	39238	Molybdenum cofactor synthesis 1 ortholog(Mocs1)	0.60
mre11	34565	meiotic recombination 11(mre11)	0.64
MRP	34686	Multidrug-Resistance like Protein 1(MRP)	0.76
mus205	47186	mutagen-sensitive 205(mus205)	0.67
Nplp4	50190	Neuropeptide-like precursor 4(Nplp4)	0.64
pic	41611	piccolo(pic)	0.35
POSH	36990	Plenty of SH3s(POSH)	0.33
pre-mod(mdg4)-K	19835678	CG44879 gene product from transcript CG44879-RB(pre-mod(mdg4)-K)	-0.08
rad50	37564	CG6339 gene product from transcript CG6339-RE(rad50)	0.55
RnrL	34392	Ribonucleoside diphosphate reductase large subunit(RnrL)	0.39
RpA-70	40972	Replication Protein A 70(RpA-70)	0.45
rpr	40015	reaper(rpr)	0.74

scaf	35505	scarface(scaf)	0.61
sda	44359	slamdance(sda)	0.38
Sox14	37822	Sox box protein 14(Sox14)	-0.46
spn-E	41919	spindle E(spn-E)	1.04
Su(Ste):CR42425	7354459	ncRNA(Su(Ste):CR42425)	-0.58
Sulf1	53437	Sulfated(Sulf1)	0.56
thetaTry	36218	thetaTrypsin(thetaTry)	-0.27
Tom	39619	Twin of m4(Tom)	0.45
Traf4	33638	TNF-receptor-associated factor 4(Traf4)	0.45
Ugt86Da	53510	CG18578 gene product from transcript CG18578-RA(Ugt86Da)	0.83
Ugt86Di	53502	CG6658 gene product from transcript CG6658-RB(Ugt86Di)	1.08
upd2	32805	unpaired 2(upd2)	0.58
wnd	40143	wallenda(wnd)	0.29
zip	38001	zipper(zip)	0.31

Supplementary Data 4. Differentially expressed genes between 10Gy irradiated *vs.* unirradiated larval male brains.

A list of 82 differentially expressed genes (42 up-regulated and 40 down-regulated) from 10Gy (B) *vs.* unirradiated (A) comparison considering a 5% false discovery rate. In the last column are represented the expression levels of each transcript calculated as Log2(B/A).

FlyBase ID	Entrez ID	Description	log2(B/A)
Aldh	34256	Aldehyde dehydrogenase(Aldh)	0.49
AOX1	41894	Aldehyde oxidase 1(AOX1)	0.85
AstC	34537	Allatostatin C(AstC)	0.42
bbx	3772670	bobby sox(bbx)	0.37
Brca2	37916	Breast cancer 2. early onset homolog(Brca2)	-0.40
CG11029	33804	CG11029 gene product from transcript CG11029-RA(CG11029)	-0.42
CG11147	33803	CG11147 gene product from transcript CG11147-RA(CG11147)	-0.36
CG11897	43450	CG11897 gene product from transcript CG11897-RB(CG11897)	0.55
CG12539	32412	CG12539 gene product from transcript CG12539-RA(CG12539)	-0.56
CG1287	40898	CG1287 gene product from transcript CG1287-RA(CG1287)	-0.66
CG13067	39785	CG13067 gene product from transcript CG13067-RA(CG13067)	0.97
CG13631	50073	CG13631 gene product from transcript CG13631-RB(CG13631)	0.53
CG15784	31461	CG15784 gene product from transcript CG15784-RB(CG15784)	0.69
CG17680	37071	CG17680 gene product from transcript CG17680-RA(CG17680)	0.37
CG18547	41452	CG18547 gene product from transcript CG18547-RA(CG18547)	1.22
CG1882	35733	CG1882 gene product from transcript CG1882-RG(CG1882)	0.50
CG30046	36334	CG30046 gene product from transcript CG30046-RC(CG30046)	-0.54
CG3008	33693	CG3008 gene product from transcript CG3008-RA(CG3008)	0.47
CG31921	319027	CG31921 gene product from transcript CG31921-RA(CG31921)	-0.56
CG32365	38876	CG32365 gene product from transcript CG32365-RB(CG32365)	0.47
CG32500	2768879	CG32500 gene product from transcript CG32500-RA(CG32500)	0.56
CG34232	5740195	CG34232 gene product from transcript CG34232-RC(CG34232)	0.54
CG3448	39094	CG3448 gene product from transcript CG3448-RB(CG3448)	0.59
CG4096	31490	CG4096 gene product from transcript CG4096-RB(CG4096)	0.41
CG43192	12798442	CG43192 gene product from transcript CG43192-RA(CG43192)	-0.64
CG5080	33299	CG5080 gene product from transcript CG5080-RB(CG5080)	0.52
CG6191	36513	CG6191 gene product from transcript CG6191-RB(CG6191)	0.39
CG8771	36397	CG8771 gene product from transcript CG8771-RB(CG8771)	-0.24
cher	42066	cheerio(cher)	0.39
Cht6	31935	CG43374 gene product from transcript CG43374-RK(Cht6)	0.51
ci	43767	cubitus interruptus(ci)	-0.41
conu	3355133	conundrum(conu)	-0.38
Corp	31764	Companion of reaper(Corp)	0.60
CR33987	3885608	ncRNA(CR33987)	0.63
CR44017	14462439	ncRNA(CR44017)	-0.63
CR44161	14462548	ncRNA(CR44161)	-0.70
CR44993	19835215	ncRNA(CR44993)	-0.55
CR45137	19835272	ncRNA(CR45137)	-0.53
CR45820	26067148	ncRNA(CR45820)	-0.54
CR45993	26067314	ncRNA(CR45993)	-0.75
CR46003	26067324	ncRNA(CR46003)	-0.33
cv-2	45280	crossveinless 2(cv-2)	0.40
Cyp310a1	35115	CG10391 gene product from transcript CG10391-RA(Cyp310a1)	-0.58
Cyp4ac2	33755	CG17970 gene product from transcript CG17970-RB(Cyp4ac2)	-0.55
fat-spondin	36919	CG6953 gene product from transcript CG6953-RA(fat-spondin)	0.45
Gadd45	35646	CG11086 gene product from transcript CG11086-RA(Gadd45)	0.75
GstT3	33047	Glutathione S transferase T3(GstT3)	0.53
GstZ2	41133	Glutathione S transferase Z2(GstZ2)	-0.32
hid	40009	head involution defective(hid)	0.77
Ilp8	39909	Insulin-like peptide 8(Ilp8)	0.44
Irbp	117419	Inverted repeat-binding protein(Irbp)	0.52
JhI-26	36819	Juvenile hormone-inducible protein 26(JhI-26)	0.76
kra	40680	krasavietz(kra)	-0.34
l(2)k09022	33960	lethal (2) k09022(l(2)k09022)	-0.33

magu	36048	CG2264 gene product from transcript CG2264-RA(magu)	**-0.48**
MRP	34686	Multidrug-Resistance like Protein 1(MRP)	**0.34**
myo	43811	myoglianin(myo)	**-0.35**
Npc1a	34358	Niemann-Pick type C-1a(Npc1a)	**-0.24**
Nrx-IV	39387	Neurexin IV(Nrx-IV)	**-0.29**
Osi14	40770	Osiris 14(Osi14)	**0.57**
pan	43769	pangolin(pan)	**-0.41**
path	39106	pathetic(path)	**-0.48**
Pten	43991	Phosphatase and tensin homolog(Pten)	**-0.43**
Pvf2	33994	PDGF- and VEGF-related factor 2(Pvf2)	**0.50**
Qtzl	318990	Quetzalcoatl(Qtzl)	**0.62**
roX1	3772376	RNA on the X 1(roX1)	**-0.50**
rpr	40015	reaper(rpr)	**0.67**
RpS9	39108	Ribosomal protein S9(RpS9)	**-0.30**
Rpt3R	41190	Regulatory particle triple-A ATPase 3-related(Rpt3R)	**0.56**
scf	38145	supercoiling factor(scf)	**0.37**
Sema-2b	246538	Semaphorin-2b(Sema-2b)	**-0.36**
snRNA:U2:34ABb	3771927	small nuclear RNA U2 at 34ABb(snRNA:U2:34ABb)	**0.62**
Sox102F	43844	CG11153 gene product from transcript CG11153-RB(Sox102F)	**-0.36**
spok	5740359	spookier(spok)	**-0.42**
Stam	34505	Signal transducing adaptor molecule(Stam)	**0.27**
Su(Ste):CR42410	7354443	ncRNA(Su(Ste):CR42410)	**-0.70**
Su(Ste):CR42415	7354448	ncRNA(Su(Ste):CR42415)	**-0.66**
Su(Ste):CR42426	7354460	ncRNA(Su(Ste):CR42426)	**-0.75**
Su(Ste):CR42430	7354464	ncRNA(Su(Ste):CR42430)	**-0.72**
Syp	42460	Syncrip(Syp)	**-0.30**
Tom	39619	Twin of m4(Tom)	**0.42**
Xrp1	42267	CG17836 gene product from transcript CG17836-RB(Xrp1)	**0.50**

Supplementary Data 5. Differentially expressed genes between 10Gy *vs.* 0.4Gy LDR irradiated larval male brains.

A list of 52 differentially expressed genes (31 up-regulated and 21 down-regulated) from 10Gy (B) *vs.* 0.4Gy LDR (C) comparison considering a 5% false discovery rate. In the last column are represented the expression levels of each transcript calculated as Log2(B/C).

FlyBase ID	ENTREZ ID	Description	Log2(B/C)
ana	35913	anachronism	**0.70**
AOX1	41894	Aldehyde oxidase 1	**0.92**
CG13067	39785	uncharacterized protein	**1.36**
CG13403	32366	uncharacterized protein	**-3.85**
CG13460	39638	uncharacterized protein	**-1.17**
CG1468	31920	uncharacterized protein	**1.26**
CG15034	31670	uncharacterized protein	**-2.51**
CG15784	31461	uncharacterized protein	**0.91**
CG18547	41452	uncharacterized protein	**1.52**
CG31789	318943	uncharacterized protein	**-5.81**
CG32198	317909	uncharacterized protein	**-2.54**
CG32447	40412	uncharacterized protein	**-0.50**
CG3819	40069	uncharacterized protein	**-4.23**
CG42326	35838	uncharacterized protein	**0.84**
CG42516	8674068	uncharacterized protein	**-5.68**
CG5080	33299	uncharacterized protein	**0.58**
CG5955	40268	uncharacterized protein	**0.66**
CG6839	40067	uncharacterized protein	**-4.24**
cher	42066	cheerio	**0.39**
Cht10	3355116	Chitinase 10	**-0.74**
Cyp28d1	33749	Cyp28d1	**0.62**
Fbp1	39566	Fat body protein 1	**2.85**
Gadd45	35646	Growth arrest and DNA damage-inducible 45	**0.90**
hid	40009	head involution defective	**0.95**
IBIN	19835457	Induced by Infection	**2.40**
Ilp8	39909	Insulin-like peptide 8	**1.61**
Irbp	117419	Inverted repeat-binding protein	**0.65**
lncRNA:CR43144	19834780	long non-coding RNA:CR43144	**1.37**
LysB	38125	Lysozyme B	**-6.82**
LysD	38127	Lysozyme D	**-4.96**
magu	36048	magu	**-0.55**
mam	36555	mastermind	**-1.42**
MRP	34686	Multidrug-Resistance like Protein 1	**0.37**
Muc68E	2768990	Mucin 68E	**-2.76**
Obp99b	43497	Odorant-binding protein 99b	**1.85**
obst-A	33022	obstructor-A	**-0.71**
path	39106	pathetic	**-0.69**
Phae1	34637	Phaedra 1	**-3.50**
Picot	36865	picot	**0.55**

Pig1	31303	Pre-intermoult gene 1	-1.31
rdog	43450	red dog mine	0.78
rpr	40015	reaper	0.84
spn-E	41919	spindle E	0.74
Su(Ste):CR42407	7354440	ncRNA	-1.98
Su(Ste):CR42410	7354443	ncRNA	-1.58
Su(Ste):CR42415	7354448	ncRNA	-1.60
Su(Ste):CR42424	7354458	ncRNA	-2.21
Su(Ste):CR42425	7354459	ncRNA	-1.37
Su(Ste):CR42426	7354460	ncRNA	-1.93
Su(Ste):CR42430	7354464	ncRNA	-1.66
vir-1	34652	virus-induced RNA 1	0.57
Wnt2	35975	Wnt oncogene analog 2	-0.65

Supplementary Data 6. RNA processing biological process.

A list of 18 differentially expressed genes between 0.4Gy LDR + 10Gy (D1-D3) and 10Gy (B1-B3) comparison that are enriched in “RNA processing” biological process obtained from DAVID functional analysis (Adjusted p-value < 0.05). For each sample is represented the normalized expression value of each gene.

Entrez-ID	FlyBase-ID	Description	Log2(D/B)	B2	B5	B6	D2	D5	D6
32568	CG8939	CG8939 gene product from transcript CG8939-RA(CG8939)	0,47	1584,662372	1681,103828	1829,256825	2442,940892	2676,731324	2182,62533
37570	CG4554	CG4554 gene product from transcript CG4554-RA(CG4554)	0,4	1990,25654	1900,37824	2158,059951	2618,085792	2987,305854	2583,78302
33960	l(2)k09022	lethal (2) k09022(l(2)k09022)	0,4	1406,015674	1381,709919	1572,234664	2057,126419	1967,410683	1837,13850
36144	CG12325	CG12325 gene product from transcript CG12325-RB(CG12325)	0,4	838,316169	737,9427338	864,4589215	1128,527987	1198,870323	998,800789
36268	CG13185	CG13185 gene product from transcript CG13185-RD(CG13185)	0,39	6725,717331	6685,058365	7523,879971	9313,247439	10566,11396	8264,66718
41891	obe	CG5205 gene product from transcript CG5205-RA(CG5205)	0,39	2935,099073	2313,626171	2626,565812	3301,316133	4208,548071	3188,79432
34656	CG6388	CG6388 gene product from transcript CG6388-RA(CG6388)	0,38	687,4589579	622,6831068	717,8096402	940,9907596	858,0279369	914,475804
42196	CG8064	CG8064 gene product from transcript CG8064-RA(CG8064)	0,36	699,3687377	683,1241307	710,091257	891,4214483	950,1475006	899,739399
45785	IleRS	Isoleucyl-tRNA synthetase(Aats-ile)	0,32	2982,738193	2660,810657	3455,520171	3888,712472	3780,850096	3921,52113
48782	Mtr4	Mtr4 helicase	0,3	1426,526962	1492,75273	1543,676646	1732,44743	1984,518602	1863,33655
39737	l(3)72Ab	lethal (3) 72Ab(l(3)72Ab)	0,28	5628,694278	5872,618556	6127,624445	7776,598789	7139,266192	6650,21214
37476	Tbp	TATA binding protein(Tbp)	-0,45	1673,324066	1291,751185	1116,078215	873,2460342	875,1358558	1047,9221
31483	RpL35	Ribosomal protein L35(RpL35)	-0,46	74160,87568	54117,20608	41225,4285	39408,42864	35943,73779	38978,6101
34751	loqs	loquacious (loq)	-0,48	3891,189732	3276,465738	2657,439345	1941,464693	2262,193288	2446,24324
3772327	snRNA:U6:96Aa	small nuclear RNA U6 at 96Aa(snRNA:U6:96Aa)	-0,56	1294,857729	1066,854352	759,4889096	611,3548394	644,8369464	604,192608
12798231	snRNA:U4atac	small nuclear RNA U4atac at 82E(snRNA:U4atac:82E)	-0,62	1985,624959	1827,286769	766,4354545	578,3086319	581,6692455	602,555230
3771927	snRNA:U2:34ABb	small nuclear RNA U2 at 34ABb(snRNA:U2:34ABb)	-0,7	3567,640713	2589,124792	2039,968687	1609,350307	1102,802778	1372,12305
