# Supplementary material for: Low dose rate γ-irradiation protects fruit fly chromosomes from double strand breaks and telomere fusions by reducing the esi-RNA biogenesis factor Loquacious
Source: Commun Biol. 2022 Sep 3;5:905. doi: 10.1038/s42003-022-03885-w (PMC9440893; doi:10.1038/s42003-022-03885-w)
Supplement: Supplementary file 5 — Reporting Summary [file 42003_2022_3885_MOESM5_ESM.pdf]

nature portfolio

Corresponding author(s): GIOVANNI CENCI COMMSBIO-22-0677A

Last updated by author(s): Jul 26, 2022

Reporting Summary

Nature Portfolio wishes to improve the reproducibility of the work that we publish. This form provides structure for consistency and transparency in reporting. For further information on Nature Portfolio policies, see our Editorial Policies and the Editorial Policy Checklist.

Statistics

For all statistical analyses, confirm that the following items are present in the figure legend, table legend, main text, or Methods section.

n/a	Confirmed	
☐	☒	The exact sample size (n) for each experimental group/condition, given as a discrete number and unit of measurement
☐	☒	A statement on whether measurements were taken from distinct samples or whether the same sample was measured repeatedly
☐	☒	The statistical test(s) used AND whether they are one- or two-sided<br>*Only common tests should be described solely by name; describe more complex techniques in the Methods section.*
☒	☐	A description of all covariates tested
☒	☐	A description of any assumptions or corrections, such as tests of normality and adjustment for multiple comparisons
☒	☐	A full description of the statistical parameters including central tendency (e.g. means) or other basic estimates (e.g. regression coefficient) AND variation (e.g. standard deviation) or associated estimates of uncertainty (e.g. confidence intervals)
☒	☐	For null hypothesis testing, the test statistic (e.g. F, t, r) with confidence intervals, effect sizes, degrees of freedom and P value noted<br>*Give P values as exact values whenever suitable.*
☒	☐	For Bayesian analysis, information on the choice of priors and Markov chain Monte Carlo settings
☒	☐	For hierarchical and complex designs, identification of the appropriate level for tests and full reporting of outcomes
☒	☐	Estimates of effect sizes (e.g. Cohen's d, Pearson's r), indicating how they were calculated

Our web collection on statistics for biologists contains articles on many of the points above.

Software and code

Policy information about availability of computer code

Data collection	RNA sequencing was carried out in single-end mode (1×75 bp) by using NextSeq500 (Illumina) with a targeted sequencing depth of about 40 million reads per sample
Data analysis	Raw data were processed with the software CASAVA v1.8.2 (Illumina) for both format conversion and de-multiplexing. Resulting reads were trimmed to remove adapter sequences using cutadapt (version 1.15) and then analyzed using the bcbio-nextgen pipeline (version 1.0.6). Specifically, reads were aligned against the Drosophila melanogaster reference genome (BDGP6) using STAR (v2.5.3a); gene abundances were estimated with Salmon (v0.9.1). Differential expression analysis at the gene level was performed using the DESeq2 R package. Genes with an adjusted p-value (FDR) <0.05 after correction for multiple testing (Benjamini-Hochberg method) were considered differentially expressed. lncRNAs polyA+ were also detected as the RNAseq library was polyA+ enriched. Finally, to analyze the functional relationships of these differentially expressed genes, a Gene Ontology (GO) functional enrichment analysis through the DAVID v. 6.8 web tool (https://david.ncifcrf.gov/,61) was performed.<br><br>The analysis of digital pictures was performed using ImageJ 1.8.0 (Image Processing and Analysis in Java).<br><br>WB Band intensities were quantified by densitometric analysis using the Image Lab 4.0.1 software (Bio-Rad)

For manuscripts utilizing custom algorithms or software that are central to the research but not yet described in published literature, software must be made available to editors and reviewers. We strongly encourage code deposition in a community repository (e.g. GitHub). See the Nature Portfolio guidelines for submitting code & software for further information.

Data

Policy information about availability of data

All manuscripts must include a data availability statement. This statement should provide the following information, where applicable:

- Accession codes, unique identifiers, or web links for publicly available datasets
- A description of any restrictions on data availability
- For clinical datasets or third party data, please ensure that the statement adheres to our policy

All the raw reads used for the analysis of differential expression have been deposited at the Sequence Read Archive (NCBI), under BioProject PRJNA747152. All supplementary materials, including the uncropped blot images, are available in the Supplementary Information and Supplementary Data files. The data that support the findings of this study are available from the corresponding author upon reasonable request.

Human research participants

Policy information about studies involving human research participants and Sex and Gender in Research.

Reporting on sex and gender	NA
Population characteristics	NA
Recruitment	NA
Ethics oversight	NA

Note that full information on the approval of the study protocol must also be provided in the manuscript.

Field-specific reporting

Please select the one below that is the best fit for your research. If you are not sure, read the appropriate sections before making your selection.

☒ Life sciences ☐ Behavioural & social sciences ☐ Ecological, evolutionary & environmental sciences

For a reference copy of the document with all sections, see nature.com/documents/nr-reporting-summary-flat.pdf

Life sciences study design

All studies must disclose on these points even when the disclosure is negative.

Sample size	Data were presented using the mean ± standard error (SE) obtained from at least three independent experiments<br>For chromosome analyses at least 150 metaphases for each condition were analyzed<br>To quantify the gH2AV foci at least 1000 cells were analyzed for each PIR.
Data exclusions	No Data were excluded for the analyses
Replication	For the cytological analyses, different techinical and biological replicates were prepared<br>RNA seq was performed on three biological replicates for conditions<br>Validation of RNA seq was carried out on biological replicates
Randomization	Third instar larvae were randomly picked from different vials for the cytological characterization of brain metaphases
Blinding	Information on treatment were kept separate from sample ID throughout the analyses

Reporting for specific materials, systems and methods

We require information from authors about some types of materials, experimental systems and methods used in many studies. Here, indicate whether each material, system or method listed is relevant to your study. If you are not sure if a list item applies to your research, read the appropriate section before selecting a response.

Materials & experimental systems

n/a	Involved in the study
☐	☒ Antibodies
☒	☐ Eukaryotic cell lines
☒	☐ Palaeontology and archaeology
☐	☒ Animals and other organisms
☒	☐ Clinical data
☒	☐ Dual use research of concern

Methods

n/a	Involved in the study
☒	☐ ChIP-seq
☒	☐ Flow cytometry
☒	☐ MRI-based neuroimaging

Antibodies

Antibodies used	anti-γH2Av (Developmental Studies Hybridoma Bank, IA 52242)<br>(FITC)-conjugated AffiniPure Donkey Anti-Mouse IgG (H+L) (1:150; Jackson ImmunoResearch)<br>the anti-Cleaved Drosophila Dcp-1 (1:50; Rabbit; Asp216 #9578; Cell Signaling Technology®)<br>AlexaFluor® 488 AffiniPure Donkey Anti-Rabbit IgG (H+L) (Jackson ImmunoResearch)<br>anti-Ku70 antibody (custom made; Agro-Bio (La Ferté St Aubin, France).<br>HRP-conjugated anti-Mouse and anti-Rabbit IgGs secondary antibody (Amersham)
Validation	Our custom made anti-Ku70 antibody was validated on ku70 null mutants. Validation of the commercial antibodies is reported in the data sheets available on the companies web sites.

Animals and other research organisms

Policy information about studies involving animals; ARRIVE guidelines recommended for reporting animal research, and Sex and Gender in Research

Laboratory animals	Drosophila melanogaster
Wild animals	This study did not involve wild type animals
Reporting on sex	We focused our transcriptomic analyses on males to avoid the confounding effects of gene expression variability of females which is known to be affected by dietary conditions during development and associated to investment into reproduction-related processes
Field-collected samples	This study did not involve samples collected from the field
Ethics oversight	No ethical approval is required for studies involving Drosophila

Note that full information on the approval of the study protocol must also be provided in the manuscript.
